# Exploring nursing students’ perceptions from nursing role function (SP-NRF) during the COVID-19 pandemic in Ardabil Province: a cross sectional study from Iran

**DOI:** 10.1186/s12912-023-01389-4

**Published:** 2023-06-26

**Authors:** Fatemeh Ahmadi, Hamidreza Shaker, Majid Eterafi, Aziz Kamran

**Affiliations:** 1 Students Research Committee, Ardabil University of Medical Sciences, Ardabil, Islamic Republic of Iran; 2 School of Medicine, Ardabil University of Medical Sciences, Ardabil, Iran

**Keywords:** Student, Perception, Nurse, Function

## Abstract

**Background:**

Perception of nursing roles among nursing students significantly influences their active engagement in nursing processes and care delivery. However, there is evidence to suggest that students’ interest in and perceptions from the nursing profession at the undergraduate level are often insufficient.

**Objective:**

This study aimed to assess nursing students’ perceptions of nursing role function and identify areas that require improvement.

**Methods:**

A cross-sectional study was conducted in 2021 among nursing students in the third- and fourth-years from three faculties in the Ardabil Province. The participants were selected through census sampling. The data were collected through interviews with the Standardized Professional Nursing Role Function (SP-NRF) questionnaire. Statistical analysis was performed using the SPSS-18 software at a significance level of less than 0.05.

**Results:**

A total of 320 nursing students participated in this study. The mean score for nursing role perception was 223.1 ± 20.3 out of 255. The results indicated significant gender differences in the mean scores of perception of the nursing role function, particularly in the supportive, professional-moral care, and professional-educational dimensions. Women scored significantly higher than men did (P < .05). Additionally, students who obtained a mean score of 19 to 20 (A) had significantly higher total scores in perception of the nursing role function than other students. Furthermore, a positive correlation was observed between students’ interest in nursing and their perceived ability with nursing role perception (r = .282, P < .01) and all its dimensions.

**Conclusion:**

Overall, nursing students demonstrated a favorable perception of nursing role function. However, their perception of mental and spiritual care was relatively weak. These findings highlight the need to review nursing education programs and incorporate the spiritual care dimension to enhance students’ understanding of and preparation for their role as nurses.

**Supplementary Information:**

The online version contains supplementary material available at 10.1186/s12912-023-01389-4.

## Introduction

The purpose of undergraduate nursing education is to prepare students to meet societal needs and possess the knowledge and skills required to provide high-quality care as professional nurses in the health system [1]. Skills and clinical training are crucial components of nursing education, requiring students to adapt to complex and changing environments and to collaborate with healthcare teams and professionals related to patient care [2]. Teamwork is a fundamental aspect of nurses’ roles and responsibilities in the health care system [3].

However, evidence suggests that nursing students may experience stress, depression, anxiety, and lifestyle habits that are detrimental to their health during their training and transition into the nursing profession [4]. This could be due to various factors, including misconceptions regarding the nursing profession. In Iran, some students enter nursing as a backup plan after failing to secure a place in medical school, which is highly competitive and offers a higher status and pay [5, 6]. This situation results in a group of students lacking proper knowledge and understanding of nursing duties [7].

Understanding nursing students’ perceptions of the profession is essential, as they can influence their professional identity and future evaluation of the profession. Studies by Arnold [4], Gagnon [3], and Porteos [8] highlight the importance of nursing students’ perceptions and experiences in shaping their professional identity and coping strategies. By examining these perceptions, educators and policymakers can develop effective strategies to support nursing students and improve the quality of nursing education and practice.

The importance of nursing students’ understanding of the profession is evident in qualitative studies on student experiences. In Arnold’s (2021) study of Arnold (2021) [4], talking about one’s own and others’ experiences and the formation of the identity of the nursing role is discussed and states that by providing simulated community nursing experiences, students’ understanding of the nurse’s role in providing care based on The facilitated community integrates theory and practice, potentially influencing future learning and patient care. Gagnon [3] identified an unexpected psychosocial element of teamwork collaboration. Porteos [8] also showed that understanding nursing students’ perspectives and insights into their coping strategies is a key element in supporting sustainable and positive learning. Internalization of nursing identity, norms, and nursing values facilitates their becoming nurses [8] and can affect the creation of professional identity and evaluation of the profession by the student as a professional in the future [9].

Therefore, policy-making is necessary to restore the historical and cultural value of the sacred nursing profession and strengthen social and political positions as one of the main pillars of health in society. One of the main policymaking principles for these measures is evidence-based cognition of nursing students’ perceptions of their role in the care process. Nursing is not only providing care and leading the care team is one of the roles of nursing, and nurses lead their patients to healthy lifestyle. Today, nursing emphasizes the importance of influencing health policy, encouraging greater investment in the nursing workforce, hiring nurses in leadership positions, and sharing nursing best practices [10]. In the process of education, by developing metacognitive functions and skills, we can reconstruct and revive the historical value of nursing, which ultimately leads to more desirable curriculum design and development of more appropriate educational strategies for nursing students [11].

The COVID-19 pandemic has presented unique challenges and opportunities for nurses and nursing students, potentially affecting their perception of the nursing profession. Nurses have experienced psychological distress while caring for COVID-19 patients, with sources of distress related to patients’ deaths, unknown disease dimensions, workplace atmosphere, professional commitments, and personal characteristics [12].

As in other days, during the Covid-19 pandemic, nurses were on the front line of care and are in close and constant contact with patients from admission to discharge [13]. Therefore, because of this care, nurses are exposed to job risks and psychological pressures and many problems such as uncertainty, feelings of anger and guilt, unpreparedness, fear and anxiety of nurses due to the painful death of a patient with COVID-19 or family breakdown and fear. They are on their way back home from being infected. As the focal point of patient care, any problem for nurses can jeopardize the entire care and patient care system [14].

Thus, the present study was conducted to evaluate nursing students’ perceptions of nurses’ role function, including the roles of professional-moral care, holistic care, education, professional, cultural, and spiritual interaction, protection, professional performance, and supportive skills during the COVID-19 pandemic in Ardabil Province, Iran. By examining the status of the students’ perceptions, we can better understand the factors that influence their views of the nursing profession and its value in promoting community health. This understanding will enable educators and policymakers to develop more effective strategies to support nursing students and improve the quality of nursing education and practice.

## Method

This descriptive-analytical cross-sectional study was conducted with third- and fourth-year nursing students in the faculties of Ardabil Province, Iran. The study protocol was approved by the Ethics Committee of the Ardabil University of Medical Sciences (IR.ARUMS.REC.1398.647). Written informed consent was obtained from all participants, and the study was conducted in accordance with the Declaration of Helsinki.

Census sampling was used to include all eligible nursing students, and 320 nursing students participated. The study used the Standardized Professional Nursing Role Function (SP-NRF) questionnaire developed by Pasyar et al. (2019) [11]. The SP-NRF questionnaire consists of 51 items organized into seven domains related to the activities and duties of nurses: professional-moral care, holistic care, educational activities, cultural and spiritual care, protective care, professional performance, and professional-supportive performance. The questionnaire was validated, with a reported reliability of 0.96.

The survey was conducted via face-to-face interviews and focused on seven areas of nurses’ role functions during the pandemic. The interviews were conducted during the students’ leisure time after providing the necessary explanations about the goals and reminding them of their voluntary participation in the study.

The demographic information collected included age, sex, year of study, and previous healthcare experience. No identifiable information was collected, and data were recorded using a questionnaire. The data were stored securely and de-identified prior to analysis.

The questionnaire scores were interpreted by calculating the total score of each participant across all seven areas. The minimum and maximum scores were 51 and 255, respectively, with higher scores indicating more positive perceptions of nurses’ role functioning during the pandemic. There was no cutoff point for the questionnaire scores, and the range of scores for each dimension of the questionnaire was not reported.

Data analysis was performed using the SPSS-18 software. Independent t-tests and one-way analysis of variance were used to compare the mean scores of the different groups at a significance level of less than 0.05. Correlation coefficients were calculated to assess the relationships between the variables.

## Results

In this descriptive-analytical cross-sectional study, 320 nursing students participated, 177 (55.3%) of whom were female. The mean age of the students was 21.6 ± 1.6 years. The students’ mean score for interest in nursing was 7.6 ± 2.1 out of 10, and their perceived ability in the nursing role was 7.2 ± 2.1. The mean score for nursing role perception was 223.1 ± 20.3 out of 255.

Table 1 presents a comparison of perceptions of nursing role function across various demographic variables including gender, marital status, indigenous status, and academic performance. The table shows the mean scores for each dimension of nursing role function and the total nursing role function perception.

**Table 1:** Comparison of Perception of Nursing Role Function by Demographic Variables

Demographic Variable		Professional Ethical Care Activities	Holistic Care Activities	Educational and Professional Activities	Protective Activities	Cultural and Spiritual Activities	Professional Skills Activities	Supportive Activities	Total Nursing Role Function Perception
**Gender**	Male	68.6 ± 6.2	57.6 ± 8.5	35.8 ± 3.2	14.0 ± 1.3	16.7 ± 3.2	13.8 ± 1.2	13.39 ± 1.7	223.1 ± 20.3
	Female	70.3 ± 5.4	59.1 ± 6.5	36.6 ± 3.5	14.2 ± 1.1	17.19 ± 2.3	14.14 ± 1.3	13.79 ± 1.5	225.5 ± 18.8
	P-value	0.01	0.06	0.04	0.08	0.14	0.08	0.03	0.01
**Marital Status**	Married	67.7 ± 7.4	57.9 ± 6.4	35.5 ± 3.9	13.4 ± 1.6	16.9 ± 2.3	13.6 ± 1.5	13.5 ± 1.7	218.8 ± 22.6
	Single	69.7 ± 5.6	58.5 ± 7.6	36.3 ± 3.4	14.1 ± 1.2	16.9 ± 2.8	14.0 ± 1.3	13.6 ± 1.6	223.8 ± 18.9
	P-value	< 0.001	< 0.001	< 0.001	0.004	0.04	< 0.001	< 0.001	< 0.001
**Indigenous Status**	Indigenous	69.0 ± 6.1	58.0 ± 7.4	36.04 ± 3.2	14.0 ± 1.36	16.8 ± 2.7	14.0 ± 1.2	13.5 ± 1.6	221.7 ± 20.3
	Non-Indigenous	70.1 ± 5.4	58.9 ± 7.5	36.5 ± 3.5	14.2 ± 1.13	17.1 ± 2.8	14.0 ± 1.4	13.6 ± 1.6	224.6 ± 20.2
	P-value	0.1	0.3	0.17	0.18	0.3	0.9	0.8	0.2
**Academic Performance**	20 − 19 (A)	72.45 ± 4.2	64.18 ± 6.3	37.54 ± 2.9	14.72 ± 0.6	18.27 ± 2.4	14.54 ± 0.52	14.27 ± 1.3	236.0 ± 16.5
	19 − 18 (B)	69.59 ± 5.9	58.10 ± 6.1	36.78 ± 2.7	14.40 ± 1.5	16.89 ± 2.9	14.18 ± 1.02	14.02 ± 1.04	224.0 ± 17.3
	18 − 17 (C)	70.72 ± 4.7	59.01 ± 7.0	36.76 ± 2.8	14.33 ± 1.07	17.00 ± 2.6	14.17 ± 1.14	13.68 ± 1.2	225.7 ± 18.2
	17 − 16 (D)	69.5 ± 5.4	58.39 ± 7.6	36.22 ± 2.8	14.10 ± 1.1	16.98 ± 2.8	14.00 ± 1.2	13.36 ± 1.4	222.8 ± 18.6
	< 16 (E)	66.63 ± 7.7	56.67 ± 8.7	34.83 ± 5.2	13.50 ± 1.5	16.75 ± 3.0	13.52 ± 1.9	13.19 ± 1.4	214.6 ± 27.1
	P-value	< 0 0.001	0.03	0.007	< 0.001	0.6	0.02	0.02	0.003

The highest scores for nursing role function were observed in the dimensions of Professional Ethical Care Activities and Holistic Care Activities, while the lowest scores were observed in the dimensions of educational, professional, and protective activities.

Table 2 displays the relationship between age, interest in being a nurse, perceived competency, and total nursing role function perception. The table also presents the mean scores for each dimension of nursing role function and their correlations with age, interest in being a nurse, and perceived competency.

**Table 2:** Relationship between age, interest to being a nurse and perceived competency with nursing role function perception

	Mean ± SD	1	2	3	4	5	6	7	8	9	10	total
Age	21.64 ± 1.6	1										
Interest to being a nurse	7.60 ± 2.1	− 0.058	1									
Perceived Competency	7.22 ± 2.1	0.078	0.333^**^	1								
Professional ethical care activities	69.5 ± 5.8	− 0.139^*^	0.281^**^	0.124^*^	1							
Holistic care activities	58.4 ± 7.5	− 0.097	0.214^**^	0.132^*^	0.678^**^	1						
Educational and professional collaborative activities	36.2 ± 3.4	− 0.035	0.254^**^	0.221^**^	0.687^**^	0.701^**^	1					
Protective activities	14.1 ± 1.2	− 0.069	0.221^**^	0.063	0.660^**^	0.493^**^	0.600^**^	1				
Cultural and spiritual activities	16.9 ± 2.8	− 0.036	0.216^**^	0.088	0.682^**^	0.762^**^	0.592^**^	0.532^**^	1			
Professional skills activities	14.02 ± 1.3	− 0.075	0.245^**^	0.169^**^	0.642^**^	0.535^**^	0.741^**^	0.548^**^	0.456^**^	1		
Supportive activities	13.6 ± 1.6	− 0.063	0.198^**^	0.137^*^	0.642^**^	0.662^**^	0.634^**^	0.478^**^	0.579^**^	0.530^**^	1	
total	223.1 ± 20.3	− 0.101	0.282^**^	0.162^**^	0.885^**^	0.909^**^	0.846^**^	0.684^**^	0.827^**^	0.714^**^	0.763^**^	1

*. Correlation is significant at the 0.05 level (2-tailed). **. Correlation is significant at the 0.01 level (2-tailed)

The results indicated a significant positive correlation between interest in being a nurse and nursing role function perception (r = .282, p < .01). Furthermore, perceived competency was positively correlated with perception of nursing role function (r = .162, p < .01). Among the dimensions of nursing role function, the strongest correlations were observed with Professional Ethical Care Activities (r = .885, p < .01) and Holistic Care Activities (r = .909, p < .01).

Additionally, it is noteworthy to investigate the relationship between age and academic year using the total score on the Nursing Role Function Perception questionnaire. Further analysis and interpretation are required to understand the potential impact of age and academic year on nursing role perceptions.

In summary, the findings of this study suggest that gender, marital status, indigenous status, and academic performance did not show substantial differences in perception of nursing role function among the participants. However, interest in being a nurse and perceived competency were positively associated with perceptions of nursing role function. The dimensions of professional ethical and holistic care activities received the highest scores, indicating their significance in the nursing role.

## Discussion

This descriptive-analytical cross-sectional study aimed to investigate the perception of nursing role function among nursing students and its relationship with demographic variables, interest in being a nurse, and perceived competency. The main findings revealed that demographic variables, including sex, marital status, indigenous status, and academic performance, did not show significant differences in nursing role function perception. However, interest in being a nurse and perceived competency were positively associated with nursing role function perception. The dimensions of Professional Ethical Care Activities and Holistic Care Activities received the highest scores, indicating their significance in the nursing role. In this discussion, we will analyze these findings in the context of nursing role function and compare them with previous studies and theoretical frameworks [15, 16].

In the results, no significant differences in perception of nursing role function across various demographic variables, including gender, marital status, indigenous status, and academic performance was seen. This finding is consistent with those of previous studies that reported no significant differences in nursing role function perception based on demographic variables. For instance, Alsyouf et al. (2018) found that gender did not significantly influence nursing students’ perceptions of their role function. Similarly, Petrou et al. (2017) reported that marital status and academic performance were not significantly associated with nursing role function perception among nursing students [17, 18].

These results suggest that nursing role function perception may be influenced by other factors, such as personal values, beliefs, and experiences, rather than by demographic characteristics [19]. This is in line with the findings of a study by Skyt [20], who reported that personal factors, such as motivation and self-efficacy, played a more significant role in shaping nursing students’ perceptions of their role function than demographic factors.

The results presented in Table 2 demonstrate a significant positive correlation between interest in being a nurse and perception of nursing role function (r = .282, p < .01). This finding is consistent with previous studies that reported a positive association between interest in nursing and nursing role function perception. For instance, a study by Quek and Shorey [21] found that nursing students with higher interest in nursing had a more positive perception of their role functions. Similarly, Johnson et al. [22] reported that interest in nursing was a significant predictor of nursing role function perception among nursing students.

Furthermore, perceived competency was positively correlated with nursing role function perception (r = .162, p < .01). This finding aligns with the literature, which suggests that nursing students who perceive themselves as more competent in their nursing role are more likely to have a positive perception of their role functions [23, 24]. Cowin et al. [25] found that perceived competency was a significant predictor of nursing role function perception, and Flinkman et al. [26] reported a positive relationship between perceived competency and nursing role function perception among nursing students.

Among the dimensions of nursing role function, the highest correlations were observed with Professional Ethical Care Activities (r = .885, p < .01) and Holistic Care Activities (r = .909, p < .01). These findings highlight the importance of these dimensions in shaping nursing students’ perceptions of their role functions. The strong correlation between these dimensions and nursing role function perception is consistent with the literature, which emphasizes the significance of professional ethical and holistic care in nursing practice [26, 27]. For example, Levett-Jones and Lathlean [19] reported that nursing students who engaged in professional ethical and holistic care activities had a more positive perception of their role function.

It is also noteworthy to investigate the relationship between age and academic year with the total score on the Nursing Role Function Perception questionnaire. Although the current study did not find a significant correlation between age and nursing role function perception, previous studies reported mixed findings regarding the relationship between age and nursing role function perception [28, 29]. For instance, a study by Alsyouf et al. [17] found that older nursing students had a more positive perception of their role function, whereas Petrou et al. [18] reported no significant relationship between age and nursing role function perception. Further research is needed to clarify the potential impact of age and academic year on nursing role perceptions. Therefore, the findings of this study suggest that interest in being a nurse and perceived competency are positively associated with perceptions of nursing role function. The dimensions of Professional Ethical Care Activities and Holistic Care Activities received the highest scores, indicating their significance in the nursing role.

Discussing the findings related to each of the nursing role performance dimensions presented in Table 2 and their implications for nursing practice and education, the dimensions are examined in separate sub-sections to provide a more detailed analysis:

### Professional ethical care activities

The strong correlation between Professional Ethical Care Activities and nursing role function perception (r = .885, p < .01) aligns with the literature, which highlights the significance of ethical care in nursing practice [30]. Nurses are expected to adhere to a code of ethics that guides their decision-making and actions in providing care to patients [31]. Numminen et al. [32] reported that nursing students who engaged in ethical care activities had a more positive perception of their role function. This suggests that fostering ethical awareness and decision-making skills among nursing students may contribute to a more positive perception of their role functions.

### Holistic care activities

The high correlation between Holistic Care Activities and nursing role function perception (r = .909, p < .01) is consistent with the literature, which emphasizes the importance of a holistic approach to patient care in nursing practice [33]. Holistic care involves addressing the physical, emotional, social, and spiritual needs of patients and recognizing the interconnectedness of these aspects in promoting overall well-being [34]. This finding is in line with the increased emphasis on holistic care in nursing education and practice, particularly during the COVID-19 pandemic [35, 36]. Papastavrou et al. [37] found that nursing students who engaged in holistic care activities had a more positive perception of their role function. This finding underscores the need for nursing education that incorporates holistic care principles and practices to enhance nursing students’ role function perception.

### Educational and professional activities

The correlation between Educational and Professional Activities and perception of nursing role function highlights the importance of collaboration and professional development in nursing practice. Nurses are expected to work closely with other healthcare professionals and engage in continuous learning to maintain and enhance their competences [38]. The positive correlation between this dimension and nursing role function perception suggests that fostering a collaborative and learning-oriented environment in nursing education may contribute to a more positive perception of nursing roles among students. In addition, the results indicated that the scores for Educational and Professional Activities were relatively lower than those for other dimensions of nursing role function. This finding may be attributed to the challenges nursing students face in balancing their academic and clinical responsibilities, particularly during the COVID-19 pandemic [39, 40].

### Protective activities

The lowest scores for nursing role function were observed for the dimensions of Protective Activities. This finding may be related to the increased workload and stress experienced by nursing students during the COVID-19 pandemic, which may have limited their ability to focus on protective aspects of care [41, 42]. However, the positive correlation between this dimension and nursing role function perception indicates that incorporating patient advocacy and safety principles into nursing education may enhance nursing students’ perceptions of their role function. Nurses play a crucial role in protecting patients from harm and ensuring that their rights are respected [43].

### Cultural and spiritual activities

The correlation between Cultural and Spiritual Activities and perception of nursing role function highlights the significance of cultural competence and spiritual care in nursing practice. Nurses are expected to provide culturally sensitive care and address patients’ spiritual needs of patients [44]. The positive correlation between this dimension and nursing role function perception suggests that promoting cultural competence and spiritual care in nursing education may contribute to a more positive perception of nursing role among students.

### Professional skills activities

The correlation between professional skill activities and nursing role function perception underscores the importance of clinical skills in nursing practice. Nurses must possess a wide range of technical and clinical skills to provide safe and effective care to patients [45]. The positive correlation between this dimension and nursing role function perception indicated that enhancing clinical skills training in nursing education may improve nursing students’ perceptions of their role function.

### Supportive activities

The correlation between Supportive Activities and perception of nursing role function emphasizes the importance of providing emotional support to patients and their families in nursing practice. Nurses play a vital role in offering comfort, empathy, and reassurance to patients and their loved ones during times of illness and distress [46]. The positive correlation between this dimension and nursing role function perception suggests that incorporating emotional support skills into nursing education may enhance nursing students’ perceptions of their role function.

The findings of this study, in relation to the perception of nursing role function among nursing students, show both similarities and differences when compared with previous research. For instance, the lack of substantial differences in nursing role function perception based on gender, marital status, indigenous status, and academic performance in this study contrasts the findings of Kumar et al. (2021), who reported significant differences in nursing role function perception based on gender and academic performance [47]. This discrepancy may be attributed to differences in sample characteristics, cultural contexts, or study designs.

In the context of the COVID-19 pandemic, the present study’s findings related to the dimensions of nursing role functioning are particularly relevant. The high scores in professional ethical and holistic care activities are consistent with the increased emphasis on ethical decision-making and holistic care during the pandemic [48]. This is supported by the study by Labrague and De Los Santos (2020), who found that nursing students’ perceptions of their professional roles during the COVID-19 pandemic were significantly influenced by their understanding of ethical and holistic care [49].

The positive correlation between interest in being a nurse and perception of nursing role function aligns with the findings of Kumar et al. (2021), who reported a significant relationship between nursing students’ interest in their profession and their perception of professional roles [47]. This finding suggests that students who are more interested in nursing are likely to have a better understanding of their professional roles and responsibilities. Moreover, the positive correlation between perceived competency and nursing role function perception is consistent with a study by Safar and Yoosefpour (2018), who found that nursing students with higher perceived competency had a better perception of their professional roles [50]. This finding highlights the importance of enhancing nursing students’ self-efficacy and competency to improve their understanding of their professional role.

The findings of this study contribute to the existing theoretical frameworks of nursing role function, such as Benner’s novice-to-expert model [51] and the Nursing Role Effectiveness Model [52]. The positive correlations between interest in being a nurse, perceived competency, and nursing role function perception support the notion that nursing students’ professional development is influenced by their motivation and self-efficacy. Furthermore, the high scores in professional ethical and holistic care activities highlight the importance of these dimensions in nursing practice, as emphasized by both Benner’s model and the Nursing Role Effectiveness Model.

## Conclusion

In conclusion, this study aimed to explore nursing students’ perceptions and experiences during the COVID-19 pandemic in the Ardabil Province, Iran, with a specific focus on nursing role function perception. The findings revealed that gender, marital status, indigenous status, and academic performance did not show substantial differences in perception of nursing role function among the participants. However, interest in being a nurse and perceived competency were positively associated with nursing role function perception.

The dimensions of Professional Ethical Care Activities and Holistic Care Activities received the highest scores, indicating their significance in the nursing role. These findings align with previous research and contribute to the existing theoretical frameworks of the nursing role function, emphasizing the integration of ethical and holistic care into nursing practice.

It is important to consider the limitations of this study, including the sample size, study design, reliance on self-reported data, and the focus on the COVID-19 pandemic. Future research should address these limitations and further explore the factors influencing nursing role function perceptions in different contexts.

Overall, this study provides valuable insights into nursing role function perceptions among nursing students during the COVID-19 pandemic. These findings can inform nursing education programs and curriculum development, highlighting the importance of nurturing students’ interest in nursing and fostering their perceived competency to enhance their engagement and satisfaction in their future nursing careers. By gaining a deeper understanding of nursing role function perceptions, educators and policymakers can better support and prepare nursing students for their professional roles in healthcare.

### Limitations of the study

Despite valuable insights gained from this study, it is essential to acknowledge its limitations. First, the sample size of 320 nursing students from Ardabil Province, Iran may not be representative of the Iranian nursing student population. Therefore, caution should be exercised when generalizing these findings to other settings or populations.

Second, the cross-sectional design of the study limits our ability to establish causal relationships between the variables. Longitudinal studies should provide a more comprehensive understanding of the changes in nursing role function perception over time.

Third, the study relied on self-reported data, which may be subject to social desirability and recall bias. Participants may have responded that they deemed socially acceptable or may not accurately remember their perceptions and experiences. Future research could employ mixed-method approaches such as interviews or observations to gather more comprehensive data.

Fourth, this study focused on nursing students’ perceptions and experiences during the COVID-19 pandemic. Although this contextual factor adds relevance and timeliness to the research, it may also limit the generalizability of the findings to non-pandemic situations. Future studies should explore nursing role function perception in different contexts to provide a more comprehensive understanding of its dynamics.

Fifth, this study did not consider other potential factors that could influence nursing role function perception, such as cultural or institutional factors. Future research should explore these factors to provide a more comprehensive understanding of this topic.

Despite these limitations, this study contributes to existing knowledge on nursing role function perception among nursing students during the COVID-19 pandemic. This study provides valuable insights into the factors that influence nursing role function perception and highlights the importance of interest in nursing and perceived competency. The findings shed light on the dimensions of nursing role function that received the highest scores, emphasizing the significance of professional ethical and holistic care activities in the nursing role.

## Electronic supplementary material

Below is the link to the electronic supplementary material.

Supplementary Material 1

## Data Availability

The datasets used and/or analyzed during the current study are available from the corresponding author upon reasonable request.
